# Histological Analysis and Gene Expression of Satellite Cell Markers in the Pectoralis Major Muscle in Broiler Lines Divergently Selected for Percent 4-Day Breast Yield

**DOI:** 10.3389/fphys.2021.712095

**Published:** 2021-08-25

**Authors:** Sara K. Orlowski, Sami Dridi, Elizabeth S. Greene, Cynthia S. Coy, Sandra G. Velleman, Nicholas B. Anthony

**Affiliations:** ^1^Department of Poultry Science, University of Arkansas, Fayetteville, AR, United States; ^2^Department of Animal Sciences, The Ohio State University, Wooster, OH, United States

**Keywords:** hyperplasia, broiler-chicken, histology, gene expression, selection

## Abstract

Muscle development during embryonic and early post-hatch growth is primarily through hyperplastic growth and accumulation of nuclei through satellite cell contribution. Post-hatch, muscle development transitions from hyperplasia to hypertrophic growth of muscle fibers. Commercial selection for breast yield traditionally occurs at ages targeting hypertrophic rather than hyperplastic growth. This has resulted in the production of giant fibers and concomitant challenges with regard to muscle myopathies. The current study investigates the impact of selection during the period of hyperplastic growth. It is hypothesized that selection for percentage breast yield during hyperplasia will result in an increased number of muscle cells at hatch and potentially impact muscle fiber characteristics at processing. This study characterizes the breast muscle histology of three broiler lines at various ages in the growth period. The lines include a random bred control (RAN) as well as lines which have been selected from RAN for high (HBY4) and low (LBY4) percentage 4-day breast yield. Post-rigor pectoralis major samples from six males of each line and age were collected and stored in formalin. The sample ages included embryonic day 18 (E18), post-hatch day 4 (d4), and day 56 (d56). The samples were processed using a Leica tissue processor, embedded in paraffin wax, sectioned, and placed on slides. Slides were stained using hematoxylin and eosin. E18 and d4 post-hatch analysis showed advanced muscle fiber formation for HBY4 and immature muscle development for LBY4 as compared to RAN. Post-hatch d56 samples were analyzed for fiber number, fiber diameter, endomysium, and perimysium spacing. Line HBY4 had the largest muscle fiber diameter (54.2 ± 0.96 μm) when compared to LBY4 (45.4 ± 0.96 μm). There was no line difference in endomysium spacing while perimysium spacing was higher for HBY4 males. Selection for percentage 4-day breast yield has impacted the rate and extent of muscle fiber formation in both the LBY4 and HBY4 lines with no negative impact on fiber spacing. The shift in processing age to later ages has exposed issues associated with muscle fiber viability. Selection during the period of muscle hyperplasia may impact growth rate; however, the potential benefits of additional satellite cells are still unclear.

## Introduction

Broiler production in the United States has increased drastically over the past 50 years as demand for poultry meat by consumers has risen. Broiler genetic progress has resulted in a bird that is faster growing, high yielding, and more efficient than the broiler from the 1950s. This has allowed for the poultry industry to meet the growing demand in a cost-effective way (Barbut et al., 2008). Unfortunately, genetic progress and changes in management and environment have resulted in concomitant challenges in the areas of meat quality, with woody breast and white striping being two myopathies that have developed in recent years (Barbut, 1996, 1997, 1998; Anthony, 1998). Woody breast is characterized as a hardening of the breast muscle at varying levels and the deposition of collagen in place of muscle fibers (Petracci et al., 2014; Sihvo et al., 2014; Tijare et al., 2016). White striping is superficial striated fat deposition that runs parallel to the muscle fibers and creates a visually unappealing filet to consumers (Kuttappan et al., 2013). Changes in management and environment, specifically changes in nutrition have been hypothesized and shown to decrease the incidence and severity of these two myopathies but at the cost of a decrease in yield (Bodle et al., 2018; Livingston et al., 2018; Meloche et al., 2018). Heritabilities have been shown to be relatively low (Bailey et al., 2015) with a majority of the variation seen in woody breast associated with non-genetic factors (Trocino et al., 2015). Novel selection methods, however, are being evaluated for their effectiveness in controlling the development of both woody breast and white striping in the industry to avoid further economic losses from the muscle myopathies. Advancements in methods of characterization may also help increase the heritability of this trait by focusing on quantitative measures instead of subjective scoring systems.

One novel selection method being evaluated at the University of Arkansas focuses on an earlier form of breast muscle development. Muscle development in a broiler occurs primarily through two different processes. The first stage of muscle development occurs during the embryonic growth period of a broiler and is called hyperplasia (Stockdale and Miller, 1987). Hyperplasia is the increase in cell, fiber, and number with cell number typically being set by hatch (Smith, 1963). Post-hatch, muscle development transitions from hyperplasia to hypertrophy, or the enlargement of tissue due to an increase in cell size (Moss, 1968; Mozdziak et al., 1997). Current commercial selection practices in broilers typically focus on ages targeting hypertrophy typically between 6 and 8 weeks of age. Therefore, an emphasis is put on an increase in fiber size and not necessarily on fiber number. While great improvements have been made in the areas of growth rate, yield, feed conversion, and disease resistance (Havenstein et al., 2003), genetic selection for hypertrophy or fiber size may be reaching a physiological limit (Mahon, 1999) as indicated by the recent development of muscle myopathies, such as white striping, and woody breast.

A majority of the breast yield at 4 days of age is going to be a result of fiber number as cells are just starting to enter into the hypertrophic growth period. Selection for 4-day percentage breast yield in broilers was hypothesized to focus not on fiber size but on fiber number. It has been shown in porcine breeds that additional fiber numbers have exhibited a positive correlation with improved meat quality (Lengerken et al., 1994; Karlsson et al., 1999; Fiedler et al., 2003; Suzuki et al., 2003). Additionally, selection at a young age may have the ability to impact the number of satellite cells, also known as adult myoblasts. Satellite cells are important in post-hatch growth and repair (Velleman et al., 2010). Satellite cells are reactivated to re-enter the cell cycle, fuse to damaged muscle fibers, and contribute their nuclei to support post-hatch growth and repair of damaged muscle fibers. The additional nuclei allow for increased protein accretion, or growth and repair of potentially damaged fibers (Fu et al., 2015). A study by Daughtry et al. (2017) found that satellite cell functionality decreased with age as they only have a certain number of divisions due to telomeric shortening. With a decrease in functionality at older ages where protein accretion is still occurring, it is possible that the satellite cells can no longer aid in repair of damaged muscle fibers resulting in the development of both woody breast and white striping. By selecting for additional satellite cells, it is possible to alleviate some of the concerns about aging satellite cells as there are more available to aid in repair.

The goal of this study was to evaluate divergently selected broiler type lines after five generations of selection for 4-day percentage breast yield. Histological changes as well as any differences in gene expression markers associated with satellite cells will be documented. These factors will be used to determine if selection at 4 days post-hatch has altered hyperplastic growth and cell number.

## Materials and Methods

### Broiler Lines

The University of Arkansas, Division of Agriculture Institutional Animal Care and Use Committee approved all live animal care and sampling utilized in this study (AUP #18083). Three broiler lines maintained and housed at the University of Arkansas were used. The first line of birds has been maintained as a random bred population that originated by mating of seven male and six female lines commercially available in the 1990s (RAN; Harford et al., 2014). From this RAN line, divergent selection was utilized to create the high (HBY4) and low (LBY4) percentage breast yield lines (Mason et al., 2020). These lines have been selected through the use of sibling selection for 4 day percentage breast yield [(Breast Wt)/(Body Wt−YolkSac)*100] for five generations. Since their creation, these lines have been maintained as closed populations and a randomly mated breeding structure is used with the avoidance of full and half siblings to decrease the rate of inbreeding accumulation.

### Sample Collection

For this study, one non-pedigreed hatch from the RAN, HBY4, and LBY4 lines was set and incubated at 37.5°C and 56% relative humidity from embryonic day 0 (E0) to embryonic day 18 (E18). At E18, they were transferred to a hatcher and hatched chicks were banded by line. Post-hatch, chicks were randomly placed by line into wood shavings litter floor pens with feed and water provided ad libitum throughout the study. Feed was formulated to meet or exceed the National Research Council (NRC) requirements with a commercial starter feed being fed from 0 to 21 days and a commercial finisher feed being feed from 22 to 56 days (NRC, 1994). At the processing age of d56, feed was removed from the birds 12 h prior to processing with access to water remaining constant.

Samples were collected at various ages throughout the embryonic and post-hatch growth period including embryonic age E18 and post-hatch d4 (selection age) and d56. These ages were selected to evaluate three periods where muscle growth and development are known to differ. At E18 and d4, samples were collected for all lines in a similar fashion. For histology, the left breast and keel were excised and stored in formalin solution until processing. At d56 post-hatch, birds were euthanized using CO_2_ gas. The birds were placed in a cooler overnight to allow for the breast muscle to complete the process of rigor mortis to avoid contraction of the collected muscle sample. After 24 h in the cooler, a small rectangular segment running parallel to the muscle fibers was cut and stored in formalin solution. At E18 and d4, samples from six males and six females were collected from each of the three lines. At d56, samples from six males and six females were collected from the HBY4 and LBY4 lines. For gene expression at E18, d4, and d56, birds were euthanized and a small section of breast muscle was immediately flash frozen in liquid nitrogen and stored at −80°C until further processing. At E18 and d4, samples were collected from six males per line. At d56, samples were collected from six males and six females from the HBY4 and LBY4 lines.

### Histology

Samples for histology at all ages were processed in a similar way. Small rectangular sections running parallel to the muscle fibers of each sample were cut and placed in plastic tissue processing cassettes in a 10% buffered formalin fixative solution (pH 7.0) at 4°C for a minimum of 17 h. Following storage in the fixative solution, muscle samples were dehydrated through a graded alcohol series previously described by Jarrold et al. (1999). Samples were then cleared in Pro-Par Clearent (Anatechm Battle Creek, MI) for 1 h with one change at 30 min. They were then infiltrated with paraffin wax at 55°C for 4 h with a single change at 1 h using a Leica TP1020 tissue processor (Leica, Nusslock, Germany). Following tissue processing, samples were then embedded in paraffin wax blocks and sectioned using a Leica microtome at a cross section of 5 μm. Four sections of each sample were adhered to Starfrost polarized slides (Mercedes Medical, Sarasota, FL) and stained using hematoxylin and Eosin Y (H&E) according to Velleman and Nestor (2004). Four images per sample were taken using an Olympus BX50 microscope (Olympus America, Center Valley, PA). At E18 and d4, 40X magnification was used and samples were compared for their stage of development. At d56, images were taken at 10X magnification and samples were evaluated for average fiber number per sample image (1,200 μm × 1,200 μm), fiber diameter, endomysium (spacing between muscle fibers), and perimysium (spacing between muscle fiber bundles) using the ImagePro^®^ software (Media Cybernetics, Bethesda, MD). For fiber number, a 200 × 200 μm grid was overlaid over each image and the number of fibers within four randomly selected squares was counted and averaged. A fiber was counted in the square if over 50% of the fiber was located within the boundaries. For fiber diameter, 30 fibers were randomly selected, measured (in μm), and averaged per sample. Thirty random distances between fibers were measured and averaged for endomysium spacing and 10 random distances between fiber bundles were measured and averaged for perimysium spacing.

### Gene Expression

Extraction of total RNA from the right breast muscle was done using Trizol reagent (Life Technologies, Grand Island, NY) using only the male samples from each line. Total RNA concentrations were determined for each sample by Take 3 Micro-Volume Plate using Synergy HT multimode microplate reader (BioTek, Winooski, VT) after DNAse treatment and purification. RNA integrity and quality were assessed by both OD260/OD280 nm absorption ratio (>1.9). For cDNA synthesis, total RNA (1 μg) was reverse transcribed using qScript cDNA Synthesis Kit (Quanta Biosciences, Gaithersburg, MD) in a 20 μl total reaction. Real-time quantitative (Applied Biosystems 7500 Real-Time PCR system) was performed using 5 μl of 10×−diluted cDNA, 0.5 μM of each forward and reverse specific primers for each gene, and SYBR Green Master Mix (Thermo Fisher Scientific, Rockford, IL) in a total 20 μl reaction (Lassiter et al., 2015; Flees et al., 2017). Oligonucleotide primers specific for paired box protein 7 (Pax-7), paired box protein 3 (Pax-3), myogenic factor 5 (Myf5), and r18S as a housekeeping gene were utilized (Table 1). The housekeeping gene was chosen as results have been constant and repeatable in several studies completed in the laboratory. Relative expressions of target genes were determined by the 2^−∆∆Ct^ method (Schmittgen and Livak, 2008). At the end of the amplification, melting curve analysis was applied using the dissociation protocol from the Sequence Detection system to exclude contamination with unspecific PCR products. The PCR products were also confirmed by agarose gel and showed only one specific band of the predicted size. Samples extracted from the E18 age, RAN line were used as a calibrator in this study.

**Table 1:** Oligonucleotide PCR/qPCR primers.

Gene	Accession number^1^	Primer sequence	Orientation	Product size, bp
PAX7	NM-205065	AGGCTCCGATGTCGAATCAG	For	55
		GCGGCGCTGCTTCCT	Rev	
PAX3	NM_204269	GCCTCACCAGCCCCAAA	For	57
		GGCTCCAGACCTCCAGTCAA	Rev	
Myf5	NM_001030363	CCTCATGTGGGCTTGCAAA	For	59
		CCTTCCGCCGGTCCAT	Rev	
r18S	AF173612	TCCCCTCCCGTTACTTGGAT	For	60
		GCGCTCGTCGGCATGTA	Rev	

*Myf5, myogenic factor 5; PAX, paired box*.

1 *Accession number refers to Genbank (NCBI)*.

### Statistical Analysis

The d56 image analysis was analyzed using a two-way ANOVA in JMP Pro 14 (SAS Institute Inc., 2010). Images were analyzed for average fiber number, fiber diameter, endomysium spacing, and perimysium spacing. The main effects analyzed were line and sex as well as the interaction between line and sex. Means were considered statistically different at a *p* < 0.05 with means being separated using the Tukey’s HSD. For each trait, gene expression results were analyzed using a two-way ANOVA using GraphPad Prism version 7.00 (GraphPad Software, La Jolla California, United States). Main effects analyzed included Line and Age as well as their interaction with means separated by Tukey’s HSD. The E18, RAN line was used as a calibrator in this study.

## Results

**Figure 1:**
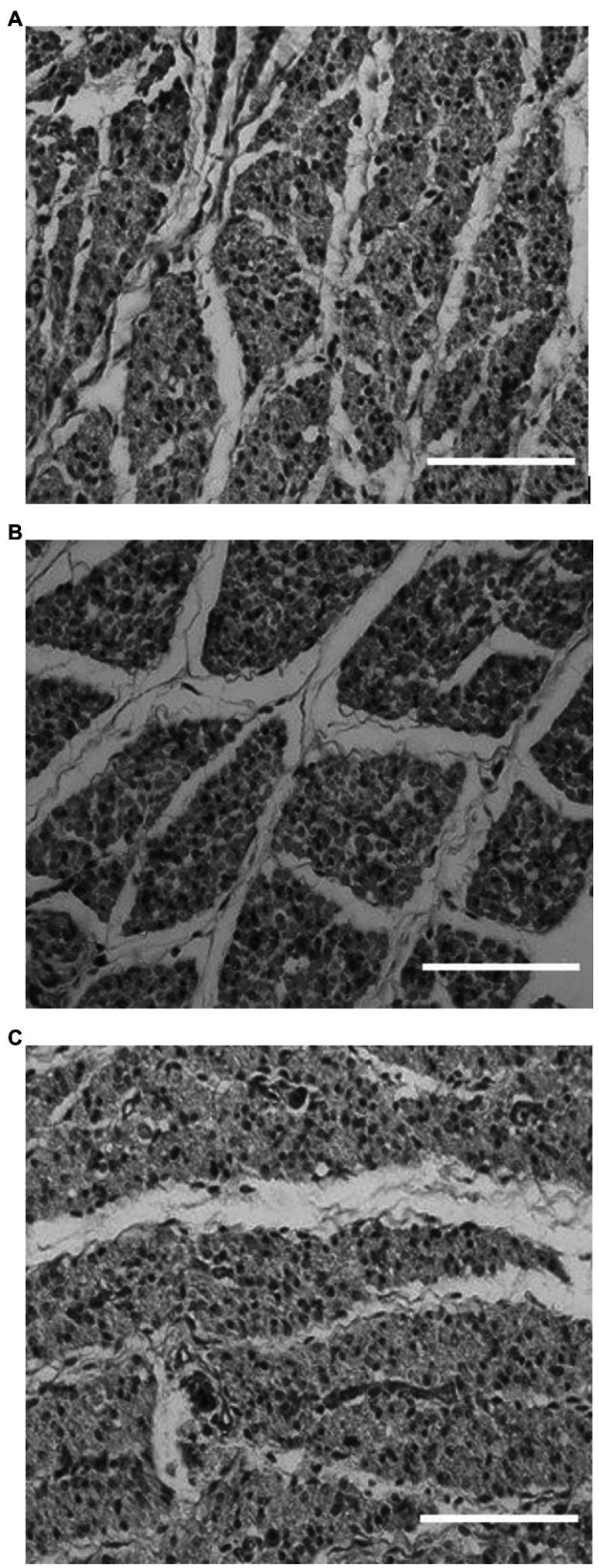
Histology images from the RAN **(A)**, HBY4 **(B)**, and LBY4 **(C)** lines at embryonic day 18 under 40X magnification (Bar = 50 μm). RAN-random bred control, BY4-high percent breast yield at 4 days post-hatch, and LBY4-low percent breast yield at 4 days post-hatch.

**Figure 2:**
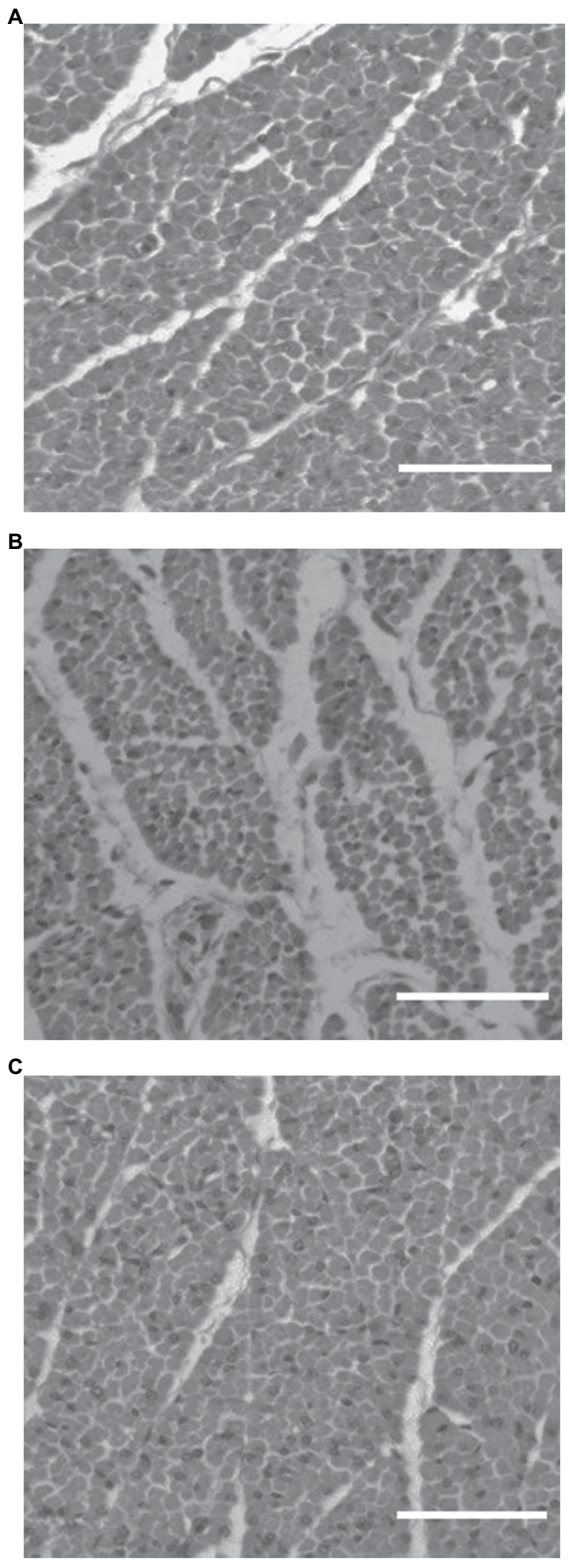
Histology images from the RAN **(A)**, HBY4 **(B)**, and LBY4 **(C)** lines at post-hatch day 4 (selection age) under 40X magnification (Bar = 50 μm). Different letters indicate significant differences at *p* < 0.05. RAN-random bred control, HBY4-high percent breast yield at 4 days post-hatch, and LBY4-low percent breast yield at 4 days post-hatch.

Quantitative image analysis was not possible for ages E18 and d4 as a result of lines being in different stages of muscle fiber formation. Images from E18 males for each line are shown in Figure 1. At this age, lines appeared to be in different stages of development with the HBY4 line showing advanced muscle fiber development by 1 to 2 days and the LBY4 line lagging behind a day when compared to the RAN line. Of the six samples taken from the HBY4 line, five of the six showed advanced muscle fiber formation when compared to the RAN while only two of the six from the LBY4 line appeared to be lagging behind in development. At d4 (selection age), it is still apparent that the HBY4 line is slightly more advanced in muscle development and growth with all of the images showing advanced muscle fiber formation. Images for the three lines at d4 are shown in Figure 2. For the LBY4 line, only three of the six samples appear similar to the RAN line in terms of fiber growth and development.

At d56, images were analyzed for fiber diameter, fiber number, endomysium spacing, and perimysium spacing. Since a line*sex interaction was not present for fiber number, fiber diameter, or endomysium only main effects are presented. Line effects were present for fiber diameter with the HBY4 line having a larger fiber than the LBY4 line. No differences were observed for the number of fibers of endomysium (fiber spacing; Table 2). A sex effect was present for fiber number in which the males had a higher fiber number than the females. No differences were observed between males and females for fiber diameter or endomysium (Table 3). A line*sex interaction was present for perimysium (muscle fiber bundle spacing). For perimysium, the HBY4 males had the greatest perimysium spacing with no difference between the LBY4 males and females from either line (Table 4).

**Table 2:** Line effects at d56 between the HBY4 and LBY4 lines^1^ for histological analysis (Mean ± SEM).

Trait	HBY4	Significance^2^	LBY4
Fiber number	17.53 ± 0.39	NS	18.02 ± 0.40
Fiber diameter	54.29 ± 1.13	^***^	45.39 ± 0.92
Endomysium	12.11 ± 0.42	NS	13.04 ± 0.33

1 *HBY4-high percent breast yield at 4 days post-hatch and LBY4-low percent breast yield at 4 days post-hatch*.

2 Significance: ^***^, highly significant (*p* < 0.001); NS, no significance.

**Table 3:** Sex effects at day 56 between males and females for histological analysis (Mean ± SE).

Trait	Males	Significance^1^	Females
Fiber number	18.38 ± 0.43	^*^	17.18 ± 0.34
Fiber diameter	47.28 ± 1.31	^***^	52.40 ± 0.98
Endomysium	12.33 ± 0.39	NS	13.04 ± 0.39

1 Significance: ^***^, highly significant (*p* < 0.001); ^*^, significant (*p* < 0.05); and NS, no significance.

**Table 4:** Line*Sex^1^ interaction for perimysium measurement (μm) at d56 (Mean ± SE)^2^.

	HBY4 – M	LBY4 – M	HBY4 – F	LBY4 – F
Perimysium	44.01 ± 2.84^a^	33.05 ± 1.60^b^	29.57 ± 1.30^b^	29.77 ± 1.35^b^

1 *HBY4-M-high 4-day breast yield males, LBY4-M-low percent breast yield males, HBY4-F-high 4-day breast yield females, and LBY4-F-low percent breast yield females*.

2 *^a–b^For each trait, groups with no common letter within a row are different at p ≤ 0.05*.

**Figure 3:**
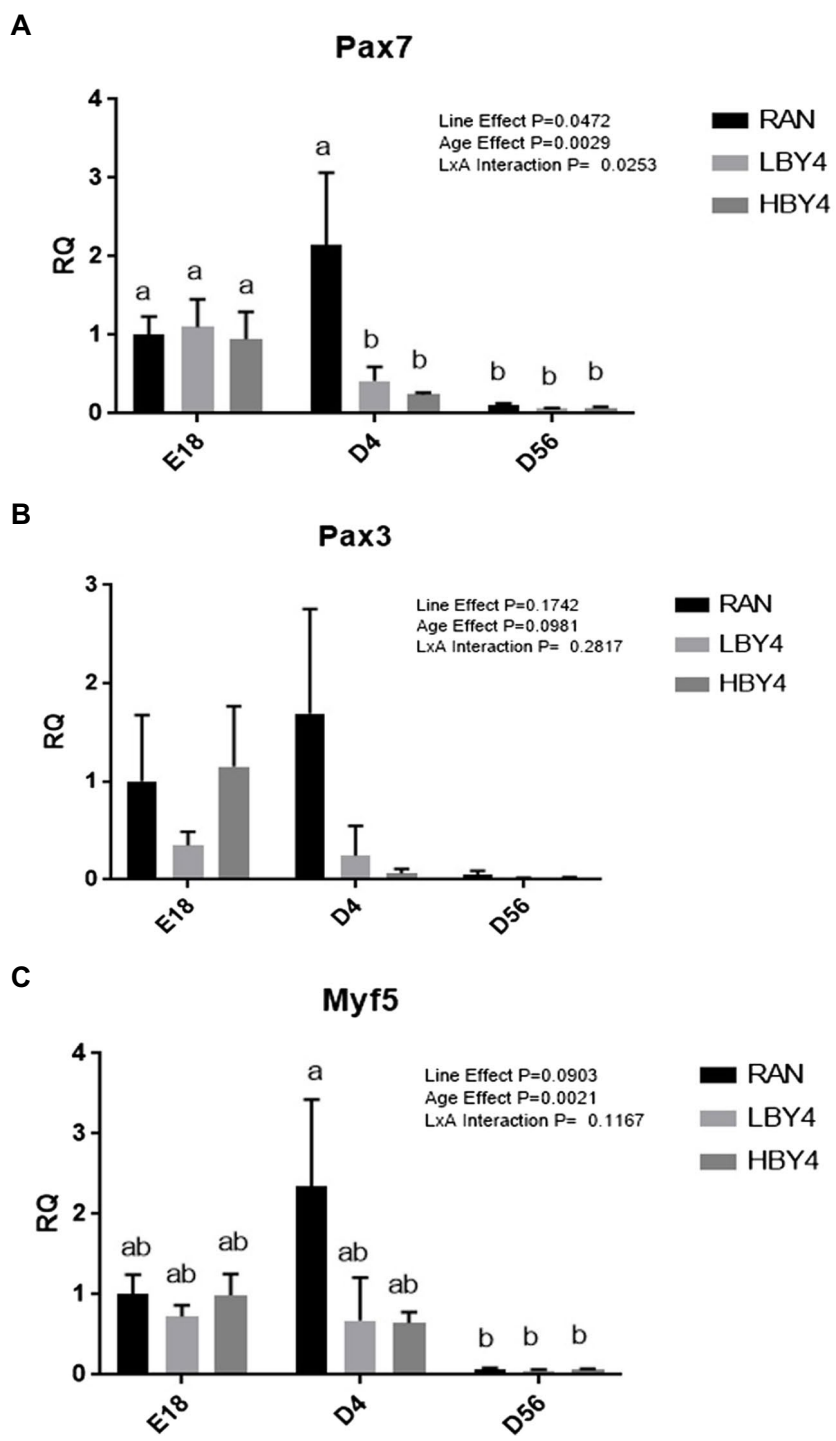
Relative gene expression (Mean ± SE) of Pax7 **(A)**, Pax3 **(B)**, and Myf5 **(C)** at embryonic day 18, d4 and d56 post-hatch for the HBY4, RAN, and LBY4 lines. Different letters indicate significant differences at *p* < 0.05. Pax7-paired box protein 7, Pax3-Paired box protein 3, Myf5-myogenic regulatory factor 5, RAN-random bred control, HBY4-high percent breast yield at 4 days post-hatch, and LBY4-low percent breast yield at 4 days post-hatch.

At E18, d4, and d56, gene expression was analyzed for Pax7, Pax3, and Myf5, all markers associated with satellite cell presence and functionality (Figure 3). For Pax7, an interaction was present for Line and Age. All lines at E18 and the RAN line at d4 had higher expression of Pax7 than the remaining groups. No differences were observed for the expression levels of Pax3 in any of the groups evaluated. For Myf5, the RAN line at d4 had the highest expression level of Myf5, while all lines at d56 had the lowest level of expression.

## Discussion

The objective of this study was to evaluate the HBY4 and LBY4 lines for differences in histological development and satellite cell associated gene expression after five generations of divergent selection for 4-day percentage breast yield. Research done in the previous generations has shown that the HBY4 line and LBY4 line do not differ in body weights throughout a 56 day grow-out period; however, their percent breast yield differs at all ages measured (Mason et al., 2020). It has remained unclear from the previous research what is driving the difference in percentage breast meat yield, whether it be from an increase in fiber number, an increase in satellite cells, or a combination of the two traits.

To better evaluate the effect of genetic selection, histology sections of the breast muscle from the LBY4, HBY4, and RAN lines were evaluated for both sexes at three different ages. The first age evaluated was E18. At E18, hyperplasia is nearly complete as the breast muscle development is in its final stage of proliferation, the satellite cell proliferation wave (Stockdale and Miller, 1987). Around this time, the number of fibers in a broiler will be at its highest (Hartley et al., 1992). Evaluation of the lines at this age showed changes in the rate of development between the lines. The HBY4 line at this age appeared to be slightly more advanced than the RAN line in its muscle fiber development. Distinct spacing between the fibers, known as the endomysium, (Aberle et al., 2012) was visible in the HBY4 line. However, in the LBY4 line, muscle fiber spacing lagged behind with a majority of the images taken at this age showing little fiber formation and only an organized mass of muscle cells. The RAN line appeared to be in between the HBY4 line and LBY4 line in terms of fiber development. It appeared that selection for a high percent breast yield at d4 has increased the rate of muscle fiber development embryonically while selection for a low percent breast yield resulted in a decrease in the rate of muscle fiber development.

The next age evaluated was the selection age of post-hatch d4. At d4, muscle development is transitioning from hyperplastic growth that occurred embryonically to hypertrophy in which cells are growing through the accumulation of protein (Moss, 1968; Mozdziak et al., 1997) from the fusion and incorporation of nuclei from the satellite cells. Satellite cells are located between the sarcolemma and basement membrane on the muscle fibers (Mauro, 1961). At this age, using H&E staining, it appeared that the HBY4 line was at a more advanced stage of development than the LBY4 and RAN lines. Images from the HBY4 line showed nuclei moving to the periphery of the muscle fiber where they will remain. The LBY4 line still showed nuclei spread throughout the fiber and was lagging slightly in the rate of development. Because the lines appeared to be at different stages of development, no numerical differences in fiber number or number of nuclei could be evaluated. It did appear that the HBY4 line entered hypertrophic growth at an earlier age than the RAN and LBY4 lines.

The final age evaluated histologically was a typical processing age of commercial broilers, post-hatch d56. At d56, only samples from the HBY4 and LBY4 lines were evaluated. Muscle sections from both lines showed healthy muscle fibers with only slight degradation of the fibers as a result of age (images not shown). Because these lines were developed from a population that was commercially available in the 1990s, they did not exhibit current muscle myopathies, such as woody breast or white striping. Typical histology samples of breast filets affected by woody breast at older ages exhibit severe fiber degeneration, fibrosis, and lipidosis (Soglia et al., 2016). Muscle sections appeared healthier, with less degeneration and fat infiltration than a modern commercial broiler breast filet that typically exhibit one or both myopathies described.

For the analysis, muscle fiber number, muscle fiber diameter, muscle fiber spacing (endomysium), and muscle fiber bundle spacing (perimysium) were evaluated. For muscle fiber number, no differences were present for fiber number between the HBY4 and LBY4 lines. As muscles grow, muscle fibers can fuse together to form larger fibers (Aberle et al., 2012). It is still possible that at a younger age, muscle fiber number differs between the HBY4 and LBY4 lines. While fiber number did not differ, fiber size was larger in the HBY4 line than the LBY4 line supporting the theory that the HBY4 line may still have a higher fiber number but those fibers have fused together resulting in a larger fiber diameter. This larger fiber diameter was also responsible for the higher yields observed in the HBY4 line. No differences were present for the endomysium, meaning the spacing of muscle fibers was similar between the lines. Visual analysis showed healthy spacing between the fibers for both lines.

A sex effect was present for both fiber number and fiber diameter. Males had a higher fiber number and a smaller fiber diameter when compared to females. In broilers, females have been shown to have a higher percentage breast meat yield, which could be a result of the larger fiber diameter and an increase in the amount of protein accretion. No difference was seen for endomysium with both males and females showing healthy fiber spacing regardless of line. An interaction was present for perimysium or the spacing between muscle fiber bundles. The HBY4 males had the largest perimysium spacing than the LBY4 males or either line of females. Results were inconsistent with a study done by An et al. (2010) in which a sex effect was present for the endomysial spacing with no effect on perimysium.

A continuing theory with the HBY4 and LBY4 lines is that selection for breast yield at an early age has the potential to impact satellite cell quantity. To evaluate this, three genes associated with satellite cells were analyzed for gene expression. Quiescent satellite cells exhibit the paired box protein 7 (Pax7) transcription factor (Seale et al., 2000). Upon activation, Pax7 was co-expressed with MyoD which is a member of the myogenic regulatory factor (MRF) family of proteins in addition to MRF 5 (Myf5). The interplay between paired box proteins and MRFs is important in the indication of self-renewal of satellite cells. Pax7 expression decreases during activation and differentiation of satellite cells (Asakura et al., 2007). MRF family proteins are the main regulators in skeletal myogenesis with Myf5 being the earliest to be expressed. The expression of the paired box protein Pax3 and Pax7 along with the expression of Myf5 is key regulators in the myogenesis process and satellite cell proliferation and activation (Francetic and Li, 2011). All three genes in combination are good indicators not only of the presence of satellite cells but also their activity as well.

This study aimed to determine what has been altered during genetic selection for 4-day relative breast yield in broilers. The gene expression levels of three key genes were the first step in determining any differences that may exist between the lines after five generations of selection. A line by age interaction was present for Pax7. The RAN line at d4 had the highest mRNA expression of PAX7 compared to the two selected lines. As selection typically occurs on the divergent lines at this age, it is possible that the expression of genes associated with satellite cells has been altered from the selection process. Looking solely at each age evaluated, no differences were observed in any of the genes measured at E18. At this age, satellite cells are still proliferating, and all three populations showed high expression levels of Pax7, Pax3, and Myf5. However, at d4, Pax7 and Pax3 appeared to be downregulated in both lines. It is possible though that for the HBY4 line, satellite cells were already active in contributing their nuclei to the fibers aiding in growth resulting in the differences in percentage breast yield when compared to the RAN line at this age. Concurrently, the LBY4 line may not have had as many satellite cells present because of selection resulting in a similarly low expression of both Pax7 and Pax3. At d56, there was no difference in expression levels between the lines. At this age period, growth is slowing and satellite cells are typically becoming mitotically quiescent, resulting in lower expression levels of genes associated with them. Evaluation of protein levels within these lines will result in a clearer understanding of the results of the selection program in relation to gene expression and will be evaluated in a future study.

## Conclusion

A divergent selection program for 4-day percentage breast yield in broilers had been implemented for five generations. After five generations of selection, it appears that the embryonic and early post-hatch development of the two broiler lines has been altered compared to their random bred control. The HBY4 line has exhibited an increased rate of muscle fiber formation while the LBY4 appeared to be lagging. At processing ages, no differences existed between the lines for fiber number while the HBY4 line exhibited a larger fiber diameter. Differences between lines for satellite cell markers were not consistent at the three ages evaluated for satellite cell markers after five generations of selection for 4-day percentage breast yield. It still remains unclear if selection for 4-day percentage breast yield has altered the number of muscle fibers or satellite cells in these populations, however is does appear that selection in the upward direction has increased both the post-hatch transition to and rate of hypertrophic growth. Future selection programs utilizing a more modern base population for line development may give better insight into the effect of selection during a period of hyperplastic on muscle myopathies, such as WB and WS.

## Data Availability Statement

The raw data supporting the conclusions of this article will be made available by the authors, without undue reservation.

## Ethics Statement

The animal study was reviewed and approved by International Animal Care and Use Committee, Protocol #18083.

## Author Contributions

SO and NA conceived and designed the study and collected the samples. SO, CC, and SV conducted the histology work. SO, EG, and SD determined gene and protein expression and analyzed the data. SO wrote the paper. All authors contributed to the article and approved the submitted version.

## Conflict of Interest

The authors declare that the research was conducted in the absence of any commercial or financial relationships that could be construed as a potential conflict of interest.

## Publisher’s Note

All claims expressed in this article are solely those of the authors and do not necessarily represent those of their affiliated organizations, or those of the publisher, the editors and the reviewers. Any product that may be evaluated in this article, or claim that may be made by its manufacturer, is not guaranteed or endorsed by the publisher.
